# From Research to Knowledge Translation: Co‐Producing Resources to Raise Awareness of Meals on Wheels in England

**DOI:** 10.1111/hex.14106

**Published:** 2024-06-14

**Authors:** Angeliki Papadaki, Paul Willis, Miranda Elaine Glynis Armstrong, Ailsa Cameron

**Affiliations:** ^1^ Centre for Exercise, Nutrition and Health Sciences, School for Policy Studies University of Bristol Bristol UK; ^2^ Centre for Research in Health and Social Care, School for Policy Studies University of Bristol Bristol UK

**Keywords:** community meals, co‐production, home‐delivered meals, infographics, knowledge translation, Meals on Wheels

## Abstract

**Background:**

Meals on Wheels (MoWs) could help adults with care and support needs continue living independently. However, many people are not aware that the service still exists in England, or that it could provide benefits beyond nutrition.

**Objective:**

Working with an existing advisory group of six people with lived experience of MoWs (an adult who uses MoWs and people who have referred a family member to MoWs), this work aimed to co‐produce knowledge translation resources (two infographics and a film) to raise awareness of MoWs and their benefits.

**Methods:**

Four participatory online workshops were held in May–July 2023, to establish perceived high‐priority themes from recent qualitative research that should be included in the resources, and preferences about message content, language, design, and how the resources should be disseminated.

**Findings:**

The most important perceived MoWs benefits that the group agreed should be included in the resources were: the importance of a nutritious meal that requires no preparation; the service's reliability/consistency; the importance of interactions in reducing social isolation, and; the ease to commence the service. The group highlighted the need for language to be nontechnical and invitational, and for images to relate to respective messages, and be inclusive of anyone who could benefit from MoWs. Several routes for dissemination were proposed, highlighting the need to disseminate to the NHS, social care organisations and community groups.

**Conclusion:**

These co‐produced resources could enhance adult social care delivery in England, as raising awareness of MoWs and their benefits could increase referral rates, so that more adults with care and support needs can benefit from the service.

**Patient or Public Contribution:**

An advisory group of people with lived experience of MoWs (users of the service and family referrers) participated in the workshops, extensively discussed the findings of earlier research, co‐produced the knowledge translation resources, and advised on the implications and future dissemination steps. The group also provided informal feedback on a draft of this manuscript.

## 1 Introduction

The number of adults in England who live with care and support needs, including older adults, and those living with functional limitations, cognitive decline, disabilities, multiple morbidities and long‐term conditions, exceeds 15 million [1, 2, 3]. Many of these adults, who wish to continue living in their homes and communities but need support to do so, could benefit from Meals on Wheels (MoWs). MoWs is a service delivering, in many countries in the world, meals to adults who are unable to leave their home to acquire ingredients, or prepare their own meals. A recent qualitative study among users of MoWs services and adults who have referred a family member to the service, recruited from four regions in England, suggested that MoWs offer four broad benefits: they provide a nutritious meal and promote overall health; they promote safeguarding and welfare; they promote independence and enhance social interactions, and; the infrastructure of the service facilitates the process of commencing the service [4]. This study also suggested that many people are not aware that the service still exists in England. For those participants who perceived ‘Meals on Wheels’ to be a generally well‐known concept, it was suggested that people do not appreciate what the service entails (i.e., that it could provide benefits beyond nutrition), until they actually need to enquire about, or use, MoWs [5]. Identifying ways to disseminate these research findings [4] to raise awareness of MoWs services and their benefits in England is, therefore, of utmost importance.

When using research findings to influence policy and practice, passive dissemination of written information (i.e., scientific papers) is often ineffective, and does not meet the preferences and needs of target audiences [6]. In contrast, an important aspect of knowledge translation is the development of dissemination materials that are attractive, easy to understand, and meet users' preferences. [6, 7] Innovative media, such as infographics and films, have been suggested to be superior to conventional knowledge translation methods (e.g., handouts, medical information sheets) for disseminating research findings [8]. Infographics, for example, present information in a logical manner, using data visualisation, text and pictures, which, according to Mayer's Cognitive Theory of Multimedia Learning, can lead to better learning and comprehension, compared to dissemination materials containing words alone [9]. The use of infographics closely relates to McGuire's Information Processing Theory [10], which proposes a matrix to explain the communication/persuasion process. This matrix consists of five input variables (source, message characteristics, channel, receiver and response target), and thirteen output variables (exposure, attention, liking, comprehension, cognitive elaboration, skill acquisition, agreement, memory, retrieval, decision making, acting on the decision, cognitive consolidation, and proselytising) [11]. Infographics fall within McGuire's second input variable (message characteristics); aiming to elicit recipients' ‘attention, comprehension, recall and action/adherence’ [11], infographics are therefore an essential means of communication to make research findings more accessible and easily understood by the general public [12]. People are likely to remember up to 6.5 times more information through an infographic than by reading text alone, rendering infographics less mentally taxing for recipients of information [13, 14]. Infographics are also essential in increasing awareness of research by both experts and nonexperts [15], and disseminating scientific research rapidly and effectively [14], and can serve as decision aids for policymakers [14, 16, 17], by eliciting ‘decision making and acting on the decision’ (two of McGuire's output variables) [11]. Because infographics, but also broadcast media, such as films, can be easily distributed through print media, embedded into websites, and shared on social media [18], they are linked to greater reach, uptake and impact of science [15]. In addition, uptake of messages has been suggested to be more effective if these knowledge translation tools (i.e., infographics and films) are co‐produced with target audiences [11].

The aim of the current work was to utilise this evidence, and findings from a recent qualitative study [4], to co‐produce, with people with lived experience of MoWs, accessible dissemination materials (two infographics and a film). We sought to develop these knowledge translation resources to raise awareness of MoWs among carers, health and social care professionals, and commissioners and policy makers, in England.

## 2 Methods

This work adapted the methods used in previous research that co‐developed health messages with the audiences for whom messages were targeted [19]. A participatory design was employed [20], underpinned by the Bristol Approach, a six‐step cyclical framework involving the following steps: ‘identification of the key issue for change, framing the issue in more detail, designing tools to address the issue, deploying the tools in the real world, orchestration to share tools and celebrate achievements, and evaluation of outcomes’ [19, 21]. The work was also informed by the ‘knowledge creation’ concept of the Knowledge to Action framework [22], which encompasses the phases of conducting research (in this case, a recent qualitative study [4, 5]), synthesising the findings (in this case, summarising the study's findings to the co‐production group), and developing tailored knowledge translation resources from the findings' synthesis (in this case, by establishing the co‐production group's preferences for the development of the resources). As this work aimed to translate knowledge, there were no ethical implications, and approval was not required from a Research Ethics Committee. All individuals who participated in the co‐production of the resources were provided with detailed information about the processes to be followed, and had the opportunity to ask questions. Those who participated in the film production provided written consent, as per University of Bristol policy.

### 2.1 Co‐Production Group

The co‐production group was an established group of people with lived experience of MoWs. One older adult who is a user of MoWs and five people who referred a family member to MoWs (females, *n* = 5; males, *n* = 1) had been recruited via their MoWs service providers from four regions in England (the North West, South West, South East and East Midlands). This group was created for the purposes of a recent MoWs study [4, 5], and have acted as advisors to MoWs research. They were compensated for the time taken to review materials and participate in the co‐production workshops, according to established policies for public contributor payment from the National Institute of Health and Care Research [23].

### 2.2 Participatory Workshops

Four online workshops, each lasting approximately 1 h, were held in May‐July 2023, using Zoom technology. The workshop activities were informed by earlier research [19], and developed further to address the specific objectives of the current work. Similar to this earlier research [19], the workshops were informal in nature and interactive. All members of the group were actively encouraged to share their thoughts and opinions on the various workshop tasks set, and discuss openly with the other members of the group.

The workshops broadly aimed to establish preferences regarding: message content (i.e., what messages should go into the resources); language (i.e., the terminology and tone of messages); the messenger of the information (i.e., who should promote or disseminate the resources), and; the mechanisms for delivering the message (i.e., how to disseminate the resources to wider audiences) [19]. The detailed plan/content of each workshop can be found in Supporting Information S1: Tables S1–S4. In summary, the group was provided with an agenda and any tasks that needed preparation before each workshop (e.g., the themes that emerged from the qualitative study whose findings would inform the knowledge translation tools [4, 5], before workshop 1). For workshops 2–4, a summary of decisions made in previous workshops was also sent to the group and agreed upon. Each workshop started with introductions; an overview of the activities for that session (and a summary of the previous session, when applicable) was provided to set expectations and remind everyone of what was aimed to be achieved. The workshops closed with summarising the decisions made, and restating how the conducted activities of that session would inform the overarching aim of the work.

As shown in Supporting Information S1: Tables S1–S4, the aim of workshop 1 was to provide the summary of findings from the qualitative study [4, 5], and gather feedback on which messages to include in the MoWs resources, and who the resources should target. The group ranked the findings in order of perceived priority for inclusion in the resources, and discussed any terms in the findings that they did not understand or like. Workshop 2 aimed to create a series of messages that would help communicate the agreed prioritised findings, with a focus on the language and tone of the messages. The aim was to develop effective messages that would aid referrals to MoWs for adults who could benefit from the service, and commissioners/policy makers to enhance services. Outcomes from the first two workshops were communicated to an experienced infographic developer. The first version of the infographic aimed at service users/referrers was developed, and sent to the research team for checking against decisions made in workshops 1–2, before being refined. The group of people with lived experience of MoWs provided feedback on this refined version of the infographic during workshop 3, considering acceptability of design, layout and images, comprehensiveness of content and language, balance between text and images, acceptability for potential diverse audiences [24], and whether the infographic reflected the decisions made by the group. This feedback informed the final refinement of this infographic by the developer, who also developed the infographic for commissioners/policy makers (using the same iterative process). Workshop 4 focused on receiving final feedback on the infographic for service users/referrers, and initial feedback on the infographic for commissioners/policy makers, before decisions were taken back to the developer to complete the final versions. Workshop 4 also aimed to identify appropriate messengers and the most appropriate mechanisms to disseminate MoWs resources to wider audiences.

With regard to the film resource specifically, feedback was sought in workshop 2 about whose voices should be represented to raise awareness of MoWs. Decisions about film content were communicated to a media company, with expertise in developing films from research studies. Film production started following workshop 2, and the film was produced and sent to participants following workshop 4.

### 2.3 Feedback Collection and Collation

The workshops were chaired by a facilitator (the first author), with another member of the research team being present to observe and take notes to cover the discussion points and contextual factors, such as group dynamics and workshop delivery, using an adapted version of a previously tested data collection form (Supporting Information S1: Tables S5–S8) [19]. Probing questions were asked, as appropriate, to clarify the group's views and allow them to provide in‐depth information. Feedback collection and collation proceeded in parallel to allow decisions made during each workshop to be communicated to the group before the next.

All workshops were recorded and transcribed by Zoom to facilitate the collation of feedback from the group, and the summary of discussion points. Collation of feedback was informed by principles of directed and conventional content analysis [25], as it was largely deductive (i.e., addressing each workshop's aims), but supplemented with any relevant inductive themes that emerged during the workshops. This was achieved by the first author reading the transcripts while listening to the recordings after each workshop, and merging this information with the observer's notes to create a single findings report of the group's views, action points, and researchers' reflections from each workshop [19, 26, 27]. The findings reports contained notes that focused on preferred content, language, design, and feedback that led to decisions about the development and dissemination of knowledge translation resources. The reports were reviewed by the research team and a summary of each workshop's findings was shared with the group to confirm accuracy of decisions. A final, de‐brief session, was also held, to obtain feedback from the group of people with lived experience of MoWs on the process of co‐producing the resources.

## 3 Findings

The feedback received is presented under three main themes, relating to the group's preferences about the development and dissemination of the MoWs knowledge translation resources, namely: message content; language and design, and; the messenger and mechanisms for disseminating the resources. During workshop 1, the group decided that the work should result in two infographics, to target two different audiences: one infographic targeted at adults who could benefit directly from MoWs (i.e., potential users of the service and carers, or family members, of adults with care and support needs), but also healthcare professionals who are considering referring an adult with care and support needs to MoWs, and; one infographic to illustrate the benefits of MoWs, targeted at people who make decisions about the service (e.g., commissioners of services and policy makers), but also healthcare providers who might not be fully aware of what MoWs entail. Further, the group suggested that the film should target all the aforementioned audiences. The presentation of the feedback reflects these decisions.

### 3.1 Preferences for Message Content

The high‐priority sub‐themes from the findings of the earlier qualitative study [4, 5], which the group agreed should be included in the resources, were: the importance of a hot, nutritious meal that requires no preparation; the reliability and consistency of service delivery; the importance of interactions in reducing isolation and loneliness, and; that the service is easy to commence. The group further identified themes that they considered important, albeit these were ranked as high‐to‐medium priority, namely: the conduct of wellbeing checks; that the service promotes independence and living in the community; the efficiency and flexibility of customer service, and; that MoWs should be recognised as an essential part of the care package that adults with care and support needs receive. The priority given to these sub‐themes was broadly similar for the development of the infographics and the film (Table 1).

**Table 1:** Priority ranking (for inclusion in the knowledge translation resources) of the main sub‐themes from the qualitative findings.

	Order of importance for sub‐theme to be included in the infographics	Order of importance for sub‐theme to be included in the film
High priority	Well known concept and a service that is easy to access	Signposting and referrals to Meals on Wheels
	Importance of a hot, nutritious meal that requires no preparation	Importance of a hot, nutritious meal that requires no preparation
	Reliability and consistency of service delivery	Reliability and consistency of service delivery
	Importance of interactions in reducing isolation and loneliness	
Highto‐medium priority	Signposting and referrals to Meals on Wheels	Well known concept and a service that is easy to access
	Carrying out welfare checks	Carrying out welfare checks
	Meals on Wheels are an essential part of the care package	Meals on Wheels are an essential part of the care package
	Promoting independence and living in the community	Importance of interactions in reducing isolation and loneliness
	Efficiency and flexibility of customer service	Promoting independence and living in the community
		Efficiency and flexibility of customer service

The group agreed that one of the infographics should focus on anyone who could refer an adult with care and support needs to MoWs, and that this should highlight that ‘the service is there to support you to support your loved one’. It was suggested that this infographic highlights signposting, that is, ‘where do I go/start to help someone get a meal?’. The group also highlighted that the messaging in all resources should challenge the preconception that MoWs are only aimed at older adults, and that the message should be inclusive for anyone in the community who might need or want to commence the service, irrespective of age, physical health or mental wellbeing. Another aspect of the service around which the group considered messaging should be inclusive, was that not all MoWs providers provide a hot meal; therefore, the messages around meal provision, in the infographics and the film, should take into account the possibility that the meals provided are chilled or frozen.

### 3.2 Preferences for Message Language and Design

The group reported that the language of the knowledge translation resources should be kept simple and nontechnical, and that words used should be ‘nonacademic’ (e.g., ‘continue living in your own home’ instead of ‘ageing in place’). They also highlighted that the language should not be patronising; for example, messages around the social benefits of MoWs should be framed factually and invitationally, and not as ‘you are lonely and therefore you need this service’. In addition, the group welcomed the incorporation in the infographics of quotations from the earlier qualitative study [4, 5], but highlighted that quotations that are framed positively would have the greatest impact (i.e., include quotations showing what benefits people get from using MoWs, instead of what would happen to them if they did not use the service).

Despite the group agreeing that perhaps the most important benefit of MoWs is the provision of a nutritious meal, they suggested that the infographic for service users and referrers focuses this message on the taste and variety of meals, but that the infographic for commissioners could use the word ‘nutritious’ or ‘healthy’. The group also highlighted that not all MoWs services provide a hot meal, so language in the knowledge translation resources around the delivery should reflect that, by indicating that ‘the meals require little or no preparation’. In addition, it was recognised that not all providers might offer wellbeing checks to the same degree, and that saying that the service will ‘check in on someone’ might not be acceptable to potential users of the service. Instead, the group suggested that the focus on the wellbeing check aspect of MoWs should be on users of the service ‘seeing a familiar face’.

The group agreed that the infographics should be dynamic, not static, and include relevant images so that target audiences can understand the messages straight away. They suggested that the infographic aimed at people who would refer an adult with care and support needs (e.g., a family member or significant other) to MoWs portrays two people in different rooms: a referrer, thinking ‘where do I start if I want to support someone get a meal?’, and a potential user of the service, thinking ‘I'd really like a hot meal and someone to say hello to’. That is, show the need for MoWs from the perspective need of these two individuals, and portray this as an illustrated story. There was also a strong preference for the inclusion of colourful images, and it was highlighted that food should be the central image in the infographic for referrers. In addition, the group reported how the images that illustrate people should be inclusive of anyone who could benefit from MoWs, including disabled and nondisabled people, and people from different ethnic minoritised groups, ages and sexes.

With regard to how signposting to services should be illustrated in the infographic for referrers, the group perceived that offering different options would be important. For example, it was deemed acceptable to provide QR codes of MoWs services for target audiences who might have higher levels of technological literacy, but also signpost referrers to calling their local authority, or adult social care services, to account for older adults or those who might be less technology competent. They suggested this could be illustrated by an image of an individual talking on the phone, or standing at a crossroads, with signposts of different routes to obtain information on how to access MoWs.

**Figure 1:**
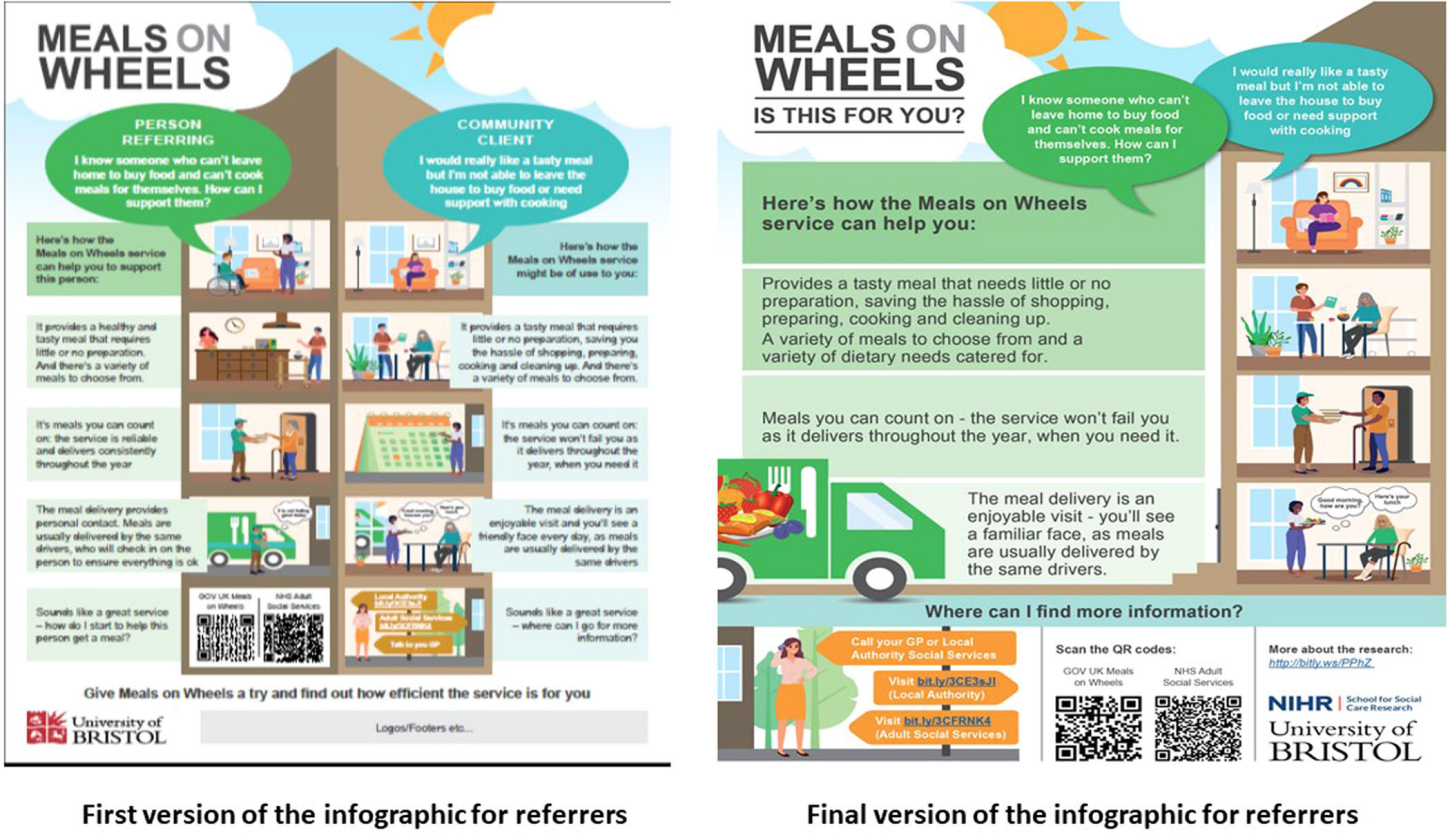
Evolution of the development of the infographic for people who could benefit from Meals on Wheels, and those who could refer an adult with care and support needs to the service, based on feedback provided in the workshops.

**Figure 2:**
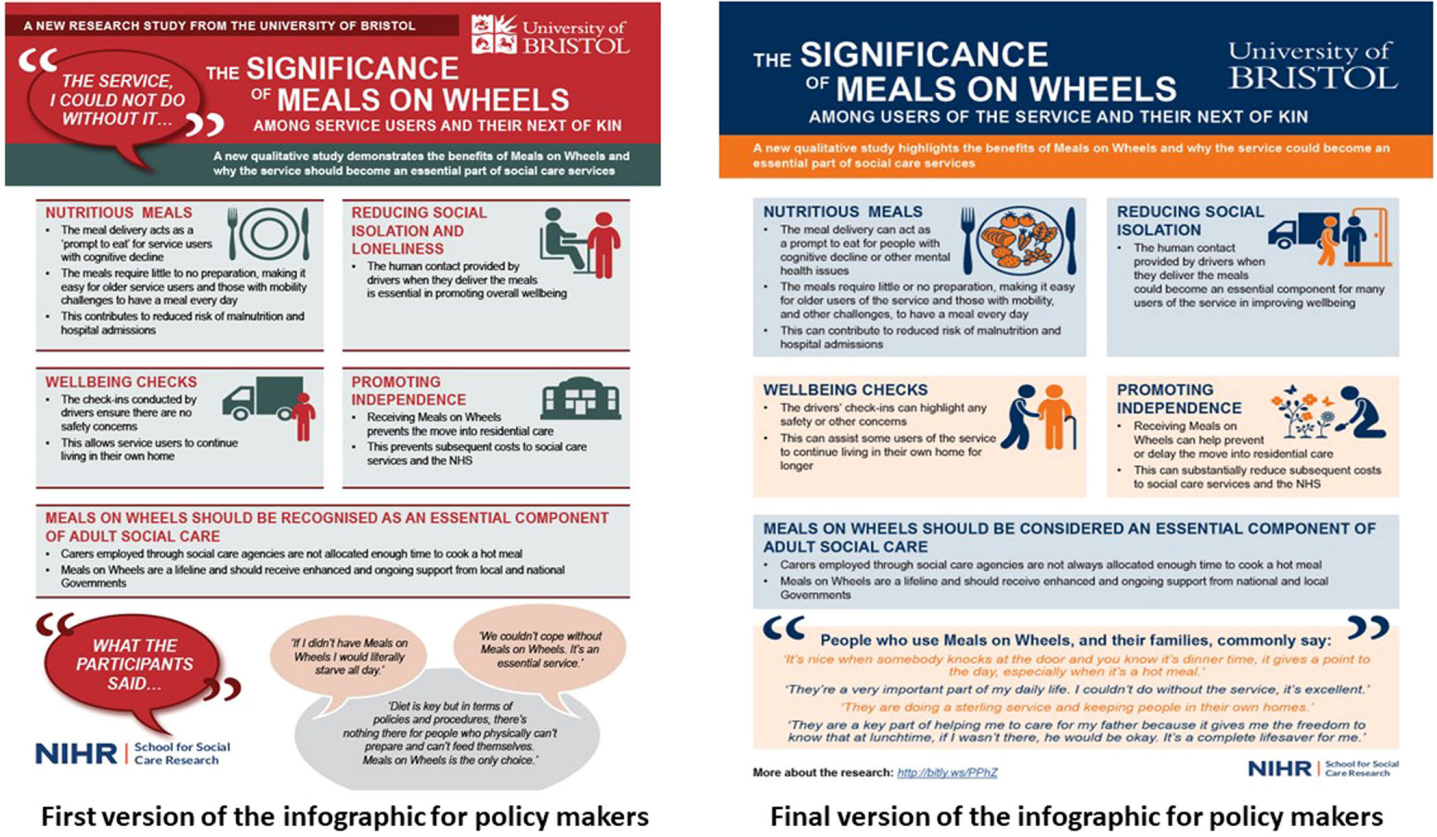
Evolution of the development of the infographic for commissioners of Meals on Wheels services, and policy makers, based on feedback provided in the workshops.

The evolution of the development of the two infographics, based on the group's feedback in the workshops, is illustrated in Figures 1 and 2, respectively. Details of the decisions made during the workshops, which informed the development of the infographics and the film, can be found in Supporting Information S1: Tables S1–S4.

### 3.3 Preferences Around the Messenger and Mechanisms of Dissemination

For the film, the group thought that having persuasive, authentic voices deliver messages, rather than academics only, would contribute best to raising awareness of MoWs services and their benefits. They suggested that the film might feature a user of MoWs discussing with an individual who referred an adult with care and support needs to the service. The final film (long version of ~7 min) touched upon all high‐priority and high‐to‐medium‐priority sub‐themes identified by the group (Table 1), featured interviews with a user of MoWs and a carer of a MoWs recipient, and included minimal contribution from the academic leading the work, who provided context and a summary of findings of the earlier qualitative study [4, 5], which informed this work. During production, the research team suggested that the film could also feature interviews with the local authority's MoWs service, and the group welcomed that. These interviews specifically covered the sub‐themes of consistency of service delivery, efficiency of customer service and signposting, but also the wider benefits of the service, as identified by the group. A shorter version of the film (~2 min) was also produced from the long version, for easier dissemination via social media channels.

The group suggested that several routes of dissemination of the knowledge translation resources would be appropriate to raise awareness of MoWs services, but that dissemination efforts should particularly focus on all contact points between the NHS (e.g., general practitioners, care coordinators of general practices, district nurses, palliative care and rehabilitation services) and social care (e.g., social workers, day care centres, lunch clubs, private care agencies for domiciliary care etc.). The need to disseminate to hospital discharge and enablement teams was particularly highlighted as a means to increase referrals to the service and reduce delayed discharges from hospital. The group also recommended to leverage community groups, community organisations, and various support groups from NHS Trusts or the voluntary sector, to raise awareness of MoWs. This included disseminating the resources to community centres, places of worship, public libraries, post offices, and even supermarkets, for the infographics, in particular, to be placed on notice boards. Finally, the infographic ‘The significance of Meals on Wheels’ was deemed important to be disseminated to health and social care professionals at a practice level, and members of the parliament at a policy level.

## 4 Discussion

Through working with people with lived experience of MoWs, this work aimed to utilise co‐production [28], and participatory research methods [20], to develop knowledge translation resources to raise awareness of MoWs. Feedback from the workshops highlight how these resources should be developed (with regard to message content, language, design), and disseminated, to raise awareness of both the existence, and the wider benefits, of this essential service, as perceived by users of MoWs services and people who have referred a family member to the service.

This work adds to the body of evidence reporting on working with people with lived experience to co‐produce infographics. Similar to the current work, a study utilising patient and public involvement to develop an infographic to promote healthy eating during pregnancy found that participants valued the use of colours and icons [29]. Further, a study that carried out iterative participatory design workshops to develop infographics with caregivers of people living with dementia to promote health self‐management and comprehension of health status, observed the need for messages and images to align [27]. Another study, which utilised participatory design methods to develop infographics to support comprehension of health information, also stressed the importance of using meaningful images that contextualise the information provided in the text [26]. Our co‐production group also highlighted the importance of accompanying messages with relevant images to aid message comprehension. For example, the group gave detailed feedback on the images provided in the first version of the infographics, which led to the incorporation, in the final versions, of images that were deemed to more closely relate to their respective messages (Figures 1 and 2). In addition, our co‐production group highlighted the need for infographics to contain accessible language, to maximise understanding and accessibility of messages, which agrees with an earlier study [27]. Of note, researchers in these earlier studies had shared prototype infographics with their participants, before refining them using participants' input. [26, 27, 29] In contrast, knowledge translation resources in the current work were co‐designed with people with lived experience of MoWs from the very beginning; apart from the research findings that would inform infographic development [4, 5], and the provision of some examples (unrelated to MoWs) to show the group what translation of research into infographics could look like, we came to the workshops ready to be steered by the co‐production group on how our infographics should be designed. Having people with lived experience take an active role in choosing message content and design could add value to the process of developing infographics, as it could enhance appeal and relevance [24].

The recent study whose methods we adapted [19] found that, consistent with the framing theory [30], focusing message content on the benefits one would gain from being physically active (‘gain framing’) could contribute to more favourable outcomes, compared with messages that focus on the harms arising from not engaging in physical activity (‘loss framing’) [19]. This agrees with feedback from our co‐production group, in that framing messages positively was considered imperative for the knowledge translation resources to raise awareness of MoWs and the service's benefits. Another study [27] utilised minimalist images of people, to avoid the potential for audiences to assign unintended meanings to their infographics with regard to age, gender and race, as suggested by earlier research [11, 26]. In contrast, our advisory group highlighted the need for the infographic for service users, and people who refer an adult with care and support needs to MoWs, to reflect the potential diversity of the people who might be using the service, and wanted to highlight that anyone could benefit from the service, irrespective of the aforementioned demographic characteristics. This highlights the importance of involving people with lived experience in the development of such resources, as feedback on the design of infographics might differ depending on the context, and what each infographic aims to achieve.

Reflection on the observations, and feedback from the group, revealed that the structured approach of the workshops worked well, and that expectations around what each workshop aimed to achieve were clear, and helped every group member to engage with the process. This navigation through the various aspects of co‐production was perceived by the group to feel well‐paced, which in turn facilitated shared understanding of each step. In the de‐brief session, members of the group further shared that they felt they could make suggestions and offer ideas, but also have counter views to others. They perceived that the workshops were held in a way that they were able to agree and disagree, and to explore ideas together with no one feeling left out. Finally, the group overwhelmingly shared that their participation in the co‐production process made them feel valued, and that they felt that the research team valued them all equally. This feedback demonstrates that co‐production is valued, suggesting that it should be embedded in the process of developing research outputs, to nurture a positive relationship between researchers and people with lived experience of the research topic.

### 4.1 Implications for Service Delivery, Policy, and Research

This work, and the co‐produced resources, could have important implications for adult social care delivery and policy in England. The infographic ‘Meals on Wheels—is this for you?’ (Figure 1), can be used by general practitioners, hospital‐based clinicians and discharge teams, and social and community carers and workers, as a resource to enhance referrals to MoWs services. It can also be used by MoWs providers as a resource to raise awareness of their services on their websites and publicity materials. The infographic ‘The significance of Meals on Wheels’ (Figure 2), which highlights the wider benefits of MoWs services, can be used by MoWs providers when they seek funding for the continuation or enhancement of their services. It can also be used by commissioners and policy makers, as an evidence‐based resource to inform decisions about reviving or reintroducing MoWs. Finally, the film can be used by MoWs providers to raise awareness of what the service entails, and its benefits, on their websites. Collectively, these knowledge translation resources could raise awareness of MoWs, inform referral processes, and support decisions around the continuation, enhancement and revival of MoWs services in England. Further, this paper contributes to understanding the process of co‐producing health and social care messages, which can benefit researchers considering embarking on developing similar knowledge translation resources worldwide.

### 4.2 Strengths and Limitations

To our knowledge, this is the first piece of work to co‐produce knowledge translation resources aiming to raise awareness of MoWs and its benefits, with people with lived experience of the service. As such, feedback from the co‐production group provides important insights on the content, language, design, and dissemination pathways that people with lived experience of MoWs consider important for such resources, to raise awareness of this essential service. The group was recruited from four different MoWs providers in England, which allowed us to consider a wide range of diversity matters, with regard to MoWs provision, when developing the resources.

Some limitations also need to be noted. The group was not ethnically diverse, and therefore the findings might not represent the preferences of the diverse population of England who could benefit from the service, with regard to how best these resources should raise awareness of MoWs amongst them. For example, it has been suggested that preferences for colours and images can vary according to cultural backgrounds [27]. Nevertheless, the group provided important insights about the diversity of MoWs recipients, and many different perspectives and ideas were shared, which informed the messages and images in the infographics. Further, the group's preference was for the second infographic to be mainly targeted to commissioners of MoWs and policy makers who make decisions about social care services. As the development of resources that meet users' preferences is an important element of knowledge translation [6, 7], this infographic should ideally have been co‐produced with these stakeholders. As such, this infographic reflects the MoWs benefits that people with lived experience of MoWs consider to be important to highlight, in order for commissioners and policy makers to enhance, or continue funding, MoWs services. Finally, the workshops were conducted online due to members of the group residing in different locations in England, and we do not know whether group dynamics would have influenced the feedback if the work had been carried out in person. Nevertheless, the workshop observations confirmed that all group members were committed to the tasks at hand, with everyone giving their views on the resources' development while respecting others' opinions.

## 5 Conclusion

Raising awareness of the existence of MoWs in England, but also of the service's wider benefits, is of utmost importance [5, 31], and could allow an increasing number of adults with care and support needs to live independently in their own homes for longer. Working with people with lived experience of MoWs, this work advances knowledge of how research evidence can be used to co‐produce knowledge translation resources that align with the preferences of this group. The feedback received highlights the importance of message content, language and design, and of utilising varied dissemination mechanisms to promote the resources. Future research should evaluate the uptake of the resources among different stakeholders and beneficiaries, and establish how they are used to raise awareness of MoWs services.

## Author Contributions

**Angeliki Papadaki:** conceptualisation, funding acquisition, writing–original draft, methodology, writing–review and editing, formal analysis, project administration, investigation, data curation. **Paul Willis:** writing–review and editing, validation, conceptualisation, investigation. **Miranda Elaine Glynis Armstrong:** writing–review and editing, validation, conceptualisation, investigation. **Ailsa Cameron:** writing–review and editing, validation, conceptualisation, investigation.

## Ethics Statement

This paper reports the process and outcomes from a knowledge translation activity. Therefore, there were no ethical considerations, and approval was not required from a Research Ethics Committee. Appropriate consent forms were completed, as per University of Bristol policy, for individuals who participated in the video production.

## Conflicts of Interest

The authors declare no conflicts of interest.

## Supporting information

Supporting information.

## Data Availability

All the materials used to co‐produce the knowledge translation resources are available in the Supporting Information of this article. Other information that supports this work is available from the corresponding author upon reasonable request. The infographics are free to download and re‐use, after reviewing a licence agreement and answering a few questions (which will allow us to follow up with individuals and organisations which use them for documenting impact purposes), from this link: https://express-licences.bristol.ac.uk/product/meals-on-wheels-awareness-raising-aids. The film is available via YouTube: https://www.youtube.com/watch?v=mO3GtnPO4E4, with a shorter version also available: https://www.youtube.com/watch?v=boUUEctKZho. An accompanying policy briefing is available from this link: https://www.bristol.ac.uk/policybristol/policy-briefings/meals-on-wheels/ For the purpose of open access, the author(s) has applied a Creative Commons Attribution (CC BY) licence to any Author Accepted Manuscript version arising from this submission.
