# Supplementary material for: From Research to Knowledge Translation: Co‐Producing Resources to Raise Awareness of Meals on Wheels in England
Source: Health Expect. 2024 Jun 14;27(3):e14106. doi: 10.1111/hex.14106 (PMC11176567; doi:10.1111/hex.14106)
Supplement: Supplementary file 1 — Supporting information. [file HEX-27-e14106-s001.docx]

**Table S1:** Workshop 1 content

**WORKSHOP 1:**

**Summary of what we will do:**

In the first workshop, we will look at the main themes that came out of the interviews with people who receive Meals on Wheels and people who refer others to the service.

**What we need to achieve by the end of the workshop:**

At the end of the workshop, we will have decided what are the most important themes and messages from the interviews, which should be included in the two infographics and film we will produce. **We will therefore discuss and decide on** **message content**.

**Before workshop 1**

1. Please look at an example of an infographic (sent to you via email)

2. Please watch two short films from other research projects (links to the films sent to you via email)

3. Look at the table that presents the summary of main findings from the interviews (at the end of this document). These are the interviews’ themes, from which you will choose the most important messages to be included in the infographics and film during the workshop. We will have these available on the day, but if you can, please have these handy during the workshop.

**During workshop 1**

Angeliki Papadaki will be facilitating the discussion. Her colleague Ailsa Cameron will also be present to keep notes. We would like to record the meeting so that we make sure we don’t miss any important points.

Please see below on what we will do during the meeting:

| **Task: Message content (i.e., what should go into the message)** | **Time (Total)** |
| --- | --- |
| *Introductions*   - One project member to welcome all public contributors to the session - Project team members to introduce themselves to the group - Public contributors to introduce themselves to the group | 5 mins (5 mins) |
| *Overview of the session*   - Purpose of the workshop and what we are hoping to achieve - Overview of the workshop activities - Public contributors seen as an equal in this process | 5 mins (10 mins) |
| *Activity – Choosing the most important messages*   - Aim: to provide the summary of findings from the study and gather feedback on which messages to include in the infographics and film - *Method(s)*:   - Chair shows summary of interview findings   - Group discussion: Conversation with participants about whether any terms in the titles of themes and sub-themes are unclear to them and they would like to change. If so, participants will be asked to explain their reasoning.   - Zoom chat: Each participant will be asked to write in the chat which themes and sub-themes are important for them to be included in the infographics and film. They will be asked to rank the perceived importance of these themes (and/or sub-themes) as a collective, and provide reasoning for their decisions. Participants will also be asked to consider the formatting of the message (e.g. length of message or personalised content), and also who the infographics and film should target.   - The Zoom chat activity will be performed twice – once for the infographics and once for the film. This will help explore whether the two knowledge translation tools should pay emphasis to different messages. A group discussion will be encouraged throughout both zoom chat activities to explore participants’ perceptions and attempt to reach a consensus on the messages to be included in the knowledge translation tools. | Summary of findings: 5 mins (15 mins)  Group discussion: 10 mins (25 mins)  Infographic zoom chat: 15 mins (40 mins)  Film zoom chat: 15 mins (55 mins) |
| *Summary and close*   - Summarise the main points of the workshop - Restate how the information generated in the workshop will feed into the overarching activity - Thank everyone for their participation | 5 mins (60 mins) |

**Please find the table of main findings on the next pages:**

**Main findings from the interviews**

| **Theme** | **Sub-theme** | **Findings** |
| --- | --- | --- |
| Setting up and accessing the service | - Well known concept and a service that is easy to access | - Easy to set up the service - ‘once you’ve ordered a few times, it’s actually quite straightforward’  - Ease of getting information about the service |
|  | - Preconceptions as a barrier to using the service | - Stereotypes exist that might prevent potential service users from accessing the service (e.g. Meals on Wheels are thought to only deliver to ‘old people’, or that they remind people of school meals)  - Importance of highlighting that the service can be provided ‘for anybody who has a need for a hot meal who physically can’t do it themselves’ |
|  | - Signposting and referrals to Meals on Wheels | - Various routes of signposting to the service (e.g. via an adult social care assessment, support/social workers and domiciliary carers)  - BUT service users most likely to be referred by a relative or next of kin |
| The importance of a nutritious meal and promoting overall health | - Importance of a hot meal that requires no preparation | - Importance of having a hot meal that does not require preparation  - Having a meal placed in front of someone acts as an incentive to eat (particularly relevant for older service users who are not keen on the hassle of preparing a hot meal, but also those with mobility issues and/ or dementia)  - Hot meal is convenient when there is lack of storage facilities (e.g. freezer) |
|  | - Meal quality (nutritional balance, taste, variety, portion size and presentation) | - Overall perception is that meals are tasty and healthy  - Meal packs contain nutritional information  - The service accounts for dietary needs (e.g. vegetarian, gluten-free, swallowing difficulties)  - Overall participants were happy with the variety of meals on offer BUT others would welcome more choices  - Overall participants were happy with the portion size BUT others would welcome slightly bigger portions  - Presentation: catering for high volumes versus improved presentation |
|  | - Appropriateness of Meals on Wheels drivers discussing and promoting physical activity | - Benefits of encouraging service users to stand up occasionally (if appropriate), go out in the garden, or move to the table to eat  - Discussing physical activity not appropriate unless drivers are trained to do so  - Taking time to discuss physical activity might prevent service users from eating their meal while it’s hot  - Physical activity should be discussed taking into account people’s abilities  - Drivers might not have the time to discuss physical activity during meal delivery rounds |
| Safeguarding and welfare | - Carrying out welfare checks | - Importance of drivers carrying out welfare checks and checking for safety hazards around service users’ homes  - Flagging concerns to service managers and service users’ next of kin, and notifying emergency services (if necessary) – peace of mind for referrers  - The relationship developed between drivers and service users allow drivers to notice any changes and flag concerns accordingly |
|  | - Meals on Wheels are an essential part of the care package | - Fundamental service for service users, *‘an important part of daily life’*  - A lifeline for referrers ‘*because otherwise I would have no life’*  - Carers officially employed through social care assessments are not allocated enough time to cook a hot meal  - An essential service for referrers who are carers themselves, supporting them with care  - Recognising food as an essential part of care and Meals on Wheels as a primary need and a statutory entitlement |
| Promoting independence and enhancing social interactions | - Reliability and consistency of service delivery | - Importance of continuity of deliveries (e.g. on national holidays, during the pandemic or in adverse weather conditions) for feeling supported and not isolated  - Importance of delivering meals at consistent times and by same teams of drivers (particularly beneficial for people living with dementia)  - BUT consistency of deliveries depends on circumstances drivers encounter in service users’ homes (e.g. an emergency encountered in one home might delay delivery to the next)  - BUT wide window of meal deliveries and time of delivery could be improved (e.g. service users who are at the beginning of a round might receive their lunch as early as 11.30 am) |
|  | - Importance of interactions in reducing isolation and loneliness | - Daily social interactions between service users and drivers who deliver the meals help tackle loneliness and give *‘a point to the day’*  - Human contact provided by the service, even if brief, is essential in promoting overall wellbeing |
|  | - Promoting independence and ‘ageing in place’ | - Importance of the service in enabling service users to stay at home and preventing the move into residential care (dual benefit for service users wishing to continue living in the community and reducing costs to social care services and the NHS)  - Importance of ageing in place for referrers’ mental health and wellbeing |
| Service operation and interactions with service users | - Efficiency and flexibility of customer service | - An efficient, responsive and accessible service – very quick to rectify problems and answer questions  - A service that cares and is ‘not just about the profit’  - Adaptable to accommodate individual needs and preferences (e.g. drivers would plate meals up for service users who are unable to do so themselves or not deliver meals that service users mentioned they did not like) or changing circumstances (e.g. possibility of cancelling a meal delivery on a specific day) |
|  | - Friendliness of drivers who deliver the meals | - Drivers are friendly and approachable  - Helping with chores around the house if needed  - Good relationships developed between drivers and service users are an important part of the support that the service provides |
|  | - Length of interactions with Meals on Wheels drivers | - Time pressures of drivers having to deliver hot meals to many service users within tight timeslots leads to brief interactions  - Brief interactions not always an issue (e.g. service users wanting to get on with their dinner)  - BUT others welcomed more time to be spent with service users as social interaction was deemed one of the most important aspects of the service (this could be achieved if more volunteers delivered meals or having smaller rounds) |
| Implications of external factors on the Meals on Wheels service | - Reliability during pandemic restrictions | - Reassurance that deliveries continued throughout the pandemic – peace of mind for both service users and referrers  - Concerns about food supplies if there was another outbreak alongside the war in Ukraine  - Concerns if Meals on Wheels service stopped due to another future lockdown, as service users would have to go into residential care (and many care homes did not accept new residents during the pandemic) |
|  | - Impact of the cost-of-living crisis | - Recent increases in the price of receiving Meals on Wheels  - BUT, overall, the service was perceived as *‘value for money’* and *‘money well spent’* due to the social care benefits it provides and the fact it’s a two-course hot meal, delivered seven days a week  - Concerns about affordability of the service for people who are struggling financially, or those who are on a state pension. |

**Table S2:** Workshop 2 content

**WORKSHOP 2:**

**Summary of Workshop 1:**

In the first workshop, we looked at the main themes/findings that came out of the interviews with people who receive Meals on Wheels and people who refer others to the service. You were asked to ‘rank’ the findings according to your preferred order of importance, so that we decide which are absolutely essential to include in the infographics and film. The collective order of perceived importance is provided at the end of this document (Tables 1-2). A summary of other decisions made and some of the thoughts raised by you is also provided after the rankings.

**Summary of what we will do in Workshop 2:**

In the second workshop, we will focus on developing the two infographics. In particular, **we will first focus on developing messages from the sub-themes that you assessed as being of high priority** **(see Table 2, sub-themes that are highlighted in green)**. If time allows, we will then focus on developing messages from the sub-themes that you assessed as being of high-to-medium priority (see Table 2, sub-themes that are highlighted in yellow).

**What we need to achieve by the end of the workshop:**

At the end of the workshop, we will have decided on how the sub-themes from the interviews that you chose to be most important, will be translated into actual messages, to be included in the two infographics. **We will therefore discuss and decide on the language of messages (e.g. the terminology and tone of the message)**.

**Before workshop 2**

1. Please look at Table 2, which presents the collective rankings of the sub-themes from the interviews (at the end of this document). We will work on developing actual messages from the high-priority (green highlight) and high-to-medium priority (yellow highlight) sub-themes. We will have these sub-themes available on the day of the workshop, but if you can, please have these handy during the workshop.

**During workshop 2**

Angeliki Papadaki will be facilitating the discussion. Her colleague Paul Willis will also be present to keep notes. We would like to record the meeting so that we make sure we don’t miss any important points.

Please see below on what we will do during the meeting:

| **Task: Language (i.e., the terminology and tone) of the message** | **Time (Total)** |
| --- | --- |
| *Overview of the session*   - Purpose of the workshop and what we are hoping to achieve - Overview of the workshop activities - Public contributors seen as an equal in this process | 5 mins (5 mins) |
| *Activity – How to communicate the messages*   - Aim: to create a series of messages that would help communicate the agreed sub-themes from workshop 1 - *Method(s)*:   - Chair shows slide with a list of the agreed sub-themes to be included in the infographics, which were decided during workshop 1.   - Participants will work together to develop effective messages that would encourage people like them to access Meals on Wheels, or policymakers to enhance the service.   - Group discussion: This activity will be positioned as a challenge, whereby participants work together to develop an effective message **from each sub-theme** that would encourage people like them to access Meals on Wheels, or encourage policymakers to enhance the service. There will be two key questions within this activity, per each message:   1) What should the message be?  2) What should the language/ tone of the message be?  We will explore these questions for each of the high-priority sub-themes first. If we have time, we will discuss the high-to-medium priority sub-themes too. Participants will be eased through these questions by the facilitator.   - - A group discussion will be encouraged throughout to explore participants’ perceptions and attempt to reach a consensus on the language of the messages to be included in the infographics.   - Film advice: Specifically for the film, we will seek feedback on whose voices will be included/ represented, and the filming location (likely in Bristol due to budget constraints but consider someone joining online). | Summary slides: 5 mins (10 mins)  Group discussion:  40 mins (50 mins)  Video advice:  5-10 (55 mins) |
| *Summary and close*   - Summarise the main points of the workshop - Restate how the information generated in the workshop will feed into the overarching activity - Thank everyone for their participation | 5 mins (60 mins) |

**Table 1. Main themes from the interviews**

| **Order of importance for theme to be included in the infographic** | **Order of importance for theme to be included in the film** |
| --- | --- |
| The importance of a hot, nutritious meal | The importance of a hot, nutritious meal |
| Checking for the welfare of Meals on Wheels recipients | Checking for the welfare of Meals on Wheels recipients |
| Setting up and accessing the service | Promoting independence and enhancing social interactions |
| Promoting independence and enhancing social interactions | Setting up and accessing the service |
| Service operation and interactions with service users | Service operation and interactions with service users |
| Implications of external factors on the Meals on Wheels service | Implications of external factors on the Meals on Wheels service |

**Table 2. Main sub-themes from the interviews**

|  | **Order of importance for sub-theme to be included in the infographic** | **Order of importance for sub-theme to be included in the film** |
| --- | --- | --- |
| **High priority** | Well known concept and a service that is easy to access | Signposting and referrals to Meals on Wheels |
|  | Importance of a hot, nutritious meal that requires no preparation | Importance of a hot, nutritious meal that requires no preparation |
|  | Reliability and consistency of service delivery | Reliability and consistency of service delivery |
|  | Importance of interactions in reducing isolation and loneliness |  |
| **High-to-medium priority** | Signposting and referrals to Meals on Wheels | Well known concept and a service that is easy to access |
|  | Carrying out welfare checks | Carrying out welfare checks |
|  | Meals on Wheels are an essential part of the care package | Meals on Wheels are an essential part of the care package |
|  | Promoting independence and living in the community | Importance of interactions in reducing isolation and loneliness |
|  | Efficiency and flexibility of customer service | Promoting independence and living in the community |
|  |  | Efficiency and flexibility of customer service |
| **Medium priority** | Preconceptions as a barrier to using the service | Preconceptions as a barrier to using the service |
|  | Friendliness of drivers who deliver the meals | Friendliness of drivers who deliver the meals |
|  | Impact of the cost-of-living crisis |  |
| **Medium-to-low priority** | Length of interactions with Meals on Wheels drivers | Length of interactions with Meals on Wheels drivers |
|  | Reliability during pandemic restrictions | Reliability during pandemic restrictions |
|  |  | Impact of the cost-of-living crisis |
| **Low priority** | Appropriateness of Meals on Wheels drivers discussing and promoting physical activity | Appropriateness of Meals on Wheels drivers discussing and promoting physical activity |

**Other decisions/ thoughts from Workshop 1:**

**For the infographics:**

- Keep language simple and non-technical, e.g. ageing in place vs. ageing in your own home; ‘safeguarding’ – use ‘non-corporate’ words

- Infographic could start with signposting – e.g. where do I go/ start to help someone get a meal?

- Infographic should be dynamic, not static, and include pictures so that people can understand the messages straight away

- We must ensure messages are transferable – not all Meals on Wheels providers offer a welfare check

- Having a separate infographic for referrers (focusing on ‘this service is there to support you to support your loved one’)

- Picture of people in two different rooms – referrer: Where do I start?; user: I’d really like to see somebody to say hello to, I’d really like a hot meal. What the referrer might want and what the service user might want. Like an illustrated story

- Messages should be inclusive for anyone in the community who might need or want the service – not mentioning age, physical and mental wellbeing

- No 1 is the nutritious and hot meal, not ‘you are lonely and you need this’. Non-patronising language

**For the film:**

- Persuasive, authentic voices, rather than academics

- A service user and a referrer talking to each other?

**Table S3:** Workshop 3 content

**WORKSHOP 3:**

**Summary of Workshop 2:**

In the second workshop, we focused on developing the infographic for potential service users and people who refer them to Meals on Wheels (‘referrers’). We developed messages from the study’s sub-themes that you assessed as being of high priority, and we decided on the language of the messages that should be included in the infographic. A summary of decisions made can be found in Table 1 (at the end of this document).

**Summary of what we will do in Workshop 3:**

In the third workshop, we will focus on providing feedback on the infographic for service users and referrers. Things to consider when providing feedback are: Acceptability of design; Layout; Visualisation of findings; Comprehensiveness of content and language; Balance between visualisation, images and text; Acceptability for different audiences; Reflection of the agreed messages.

**What we need to achieve by the end of the workshop:**

At the end of the workshop, we will have decided on any changes to make to the infographic for service users and referrers.

**Before workshop 3**

1. Please look at the infographic for service users and referrers (attached to the email message). We will have this available on the day of the workshop, but if you can, please have this handy during the workshop.

**During workshop 3**

Angeliki Papadaki will be facilitating the discussion. Her colleague Paul Willis will also be present to keep notes. We would like to record the meeting so that we make sure we don’t miss any important points.

Please see below on what we will do during the meeting:

| **Task: Feedback on the infographic** | **Time (Total)** |
| --- | --- |
| *Overview of the session*   - Purpose of the workshop and what we are hoping to achieve - Overview of the workshop activities - Public contributors seen as an equal in this process | 5 mins (5 mins) |
| *Activity – Feedback on the infographic*   - Aim: To present the infographic for service users and referrers and gather feedback on it - *Method(s)*:   - Facilitator shows infographic to participants   - Group discussion: Participants will be asked about three things they like, and three things that they would like to see improved (or that they don’t like) in the infographic. Participants will be asked to explain their reasoning.   - Group discussion: We will ensure we cover, and receive feedback on, the following aspects:   - Acceptability of design   - Layout   - Visualisation of findings (images)   - Comprehensiveness of content and language   - Balance between images and text   - Acceptability for different audiences   - Does the infographic reflect what we agreed on in workshop 2?   - A group discussion will be encouraged throughout to explore participants’ perceptions and attempt to reach a consensus on the final refinement of the infographic. | Infographic: 5 mins (10 mins)  Group discussion 1: 25 mins (35 mins)  Group discussion (infographic 1): 20 mins (55 mins) |
| *Summary and close*   - Summarise the main points of the workshop - Restate how the information generated in the workshop will feed into the overarching activity - Thank everyone for their participation | 5 mins (60 mins) |

**Table 1: Summary of decisions made in workshop 2**

|  | **Order of importance for sub-theme to be included in the infographic** | **Group suggestions** | **Which infographic should this be included into** |
| --- | --- | --- | --- |
| **High priority** | Importance of a hot, nutritious meal that requires no preparation | Not all services offer a hot meal (some offer chilled or frozen) so we need to keep this inclusive. Even the services that offer a hot meal for lunch, offer a chilled meal for dinner (e.g. sandwiches, in addition to the hot lunch). Perhaps ‘hot or cold tasty meals’**.**  The suggestion was to focus on the healthiness (for referrers and commissioners) and taste (for service users and referrers) of the meal, and highlight that the meal requires little or no preparation (it takes away the burden of: shopping, preparing, cooking and cleaning up; for both service users’ and referrers’ infographics). Also, to highlight that there is a variety of meals to choose from (for both service users’ and referrers’ infographics).  The term ‘nutritious’ can be used for the commissioners’ infographic. For the service users’ and referrers’ infographic, the words ‘tasty’ and/or ‘delicious’ should be used.  Image of food on wheels i.e. a bowl of soup on a set of wheels, possibly show choice. | Both infographics |
|  | Reliability and consistency of service delivery | Concept here is that people can count on the service. Participants suggested ‘meals you can count on’ or ‘the service won’t fail you’. | Infographic for service users and referrers only |
|  | Importance of interactions in reducing isolation and loneliness | Considering that it’s usually the same driver delivering to a service user, highlight the relationship developed with the same driver – which links to the consistency of delivery above)  For users – could emphasise ‘enjoy the visit’. For referrers, ‘personal contact’.  Illustration: two people enjoying chit chatting (maybe an intergenerational image) | Both infographics |
|  | Well known concept and a service that is easy to access | Combine this sub-theme with the sub-theme ‘Signposting and referrals to Meals on Wheels‘ (see below) and keep this to the bottom of the infographic (see table on page 1)  Concept: here’s what Meals on Wheels can do for you; if you want to access the service, here’s how’  Note: participants suggested the following routes to getting information: leaflet, telephone someone in the local authority, leaders in the community (religious groups etc), general practitioners. These might be viable ways, but as far as we know, the only ‘official’ way is through a governmental link (https://www.gov.uk/meals-home, where you enter your postcode, leading to local authority information). Perhaps the infographic can include a QR code to this website, at least for the referrers’ infographic?  Participants highlighted that service users might not be technology-literate, and the QR code might not work for them, so ‘call your local authority’ might be more appropriate for the service users’ infographic.  Suggested illustration of someone standing at a crossroads, with signposting of different routes to get information (phone, computer etc). Could use image of smiling face talking on phone for the user infographic. | Infographic for service users and referrers only (but the QR code might be useful for general practitioners who want to refer someone to the service) |
| **High-to-medium priority** | Signposting and referrals to Meals on Wheels | Combine this sub-theme with the sub-theme ‘Well known concept and a service that is easy to access’ and keep this to the bottom of the infographic (see table on page 1) | Infographic for service users and referrers only (but the QR code might be useful for GPs who want to refer someone to the service) |
|  | Carrying out welfare checks | Not all services carry out welfare checks to the same degree, so perhaps not appropriate to use this term. What all services do is check on service users.  Participants suggested to use the term ‘do they seem all right’ for the service users’ infographic, and ‘keeping an eye on recipients of meals’ for the referrers’ infographic.  The ‘checking-in’ aspect might be more important to referrers than users, as no one would like to be ‘checked’. For users it might be ‘a friendly face’.  This could be illustrated by a picture of the driver calling the service manager to report that something is not right.  The term ‘welfare checks’ can be used for the commissioners’ infographic. | Both infographics |
|  | Meals on Wheels are an essential part of the care package |  | Infographic for commissioners only |
|  | Promoting independence and living in the community | Keep language simple - ‘continue living in your own home’ instead of ‘ageing in place’.  Cost savings to social care and NHS services will be more relevant to commissioners than referrers. ‘Helping to promote independence’ was more important for referrers than service users. | Infographic for referrers and commissioners only |
|  | Efficiency and flexibility of customer service | Participants mentioned this can be skipped from the infographic as they realise space is tight. If this sub-theme is included, it could be in the sense of ‘you’ll find out once you start using the service’, or ‘give it a try and find out how efficient the service is for you’. | Infographic for service users and referrers only |

**Table S4:** Workshop 4 content

**WORKSHOP 4:**

**Summary of Workshop 3:**

In the third workshop, we discussed the first version of the infographic for potential service users and people who refer them to Meals on Wheels. A summary of decisions made can be found at the end of this document.

**Summary of what we will do in Workshop 4:**

In the fourth workshop, we will focus on providing feedback on the next-to-final version of the infographic for service users and referrers. We will also have time to discuss feedback on the first version of the infographic for commissioners of services. Things to consider when providing feedback are: Acceptability of design; Layout; Visualisation of findings; Comprehensiveness of content and language; Balance between visualisation, images and text; Acceptability for different audiences; Reflection of the agreed messages.

Finally, we will discuss how best to disseminate the infographics to wide audiences (and to what audiences).

**What we need to achieve by the end of the workshop:**

At the end of the workshop, we will have decided on any **essential** changes to make to the infographic for service users and referrers, and on any changes to make to the infographic for commissioners. We will also have decided on audiences that should be made aware of the infographics and film, and how best to let them know about them.

**Before workshop 4**

1. Please look at the infographic for service users and referrers (attached to the email message). We will have this available on the day of the workshop, but if you can, please have this handy during the workshop.

2. Please look at the infographic for commissioners (attached to the email message). We will have this available on the day of the workshop, but if you can, please have this handy during the workshop.

**During workshop 4**

Angeliki Papadaki will be facilitating the discussion. Her colleague Miranda Armstrong will also be present to keep notes. We would like to record the meeting so that we make sure we don’t miss any important points.

Please see below on what we will do during the workshop:

| **Task: Feedback on the infographics and how (and to whom) to disseminate** | **Time (Total)** |
| --- | --- |
| *Overview of the session*   - Purpose of the workshop and what we are hoping to achieve - Overview of the workshop activities - Public contributors seen as an equal in this process | 5 mins (5 mins) |
| *Activity – Feedback on the infographic*  Aim 1: To present the infographic for service users and referrers and gather final feedback on it   - *Method(s)*:   - Facilitator shows infographic to participants   - Group discussion: Participants will be asked to give feedback on this next-to-final version, and note any final changes that they consider essential to be made. Participants will be asked to explain their reasoning.   Aim 2: To present the infographic for commissioners and gather feedback on it   - *Method(s)*:   - Facilitator shows infographic to participants   - Group discussion: Participants will be asked about three things they like, and three things that they would like to see improved (or that they don’t like) in the infographic. Participants will be asked to explain their reasoning.   - Group discussion: We will ensure we cover, and receive feedback on, the following aspects:   - Acceptability of design   - Layout   - Visualisation of findings (images)   - Comprehensiveness of content and language   - Balance between images and text   - Acceptability for different audiences   - Does the infographic reflect what we agreed on in workshop 2?   - A group discussion will be encouraged throughout to explore participants’ perceptions and attempt to reach a consensus on the final refinement of the infographics.   *Activity – Identifying the audiences and ways to share the knowledge translation resources*    Aim 3: To discuss who we should send the infographics and film to, and how   - - Group discussion: This activity will focus on two key questions:   - 1) Who should the infographics and film be sent to?   - 2) How should the infographics and film be promoted? | Aim 1 with group discussion: 10 mins (15 mins)  Aim 2 with group discussion: 20 mins (35 mins)  Aim 3 with group discussion: 20 mins (55 mins) |
| *Summary and close*   - Summarise the main points of the workshop - Restate how the information generated in the workshop will feed into the overarching activity - Thank everyone for their participation | 5 mins (60 mins) |

**Action points/ decisions from Workshop 3:**

Infographic for Meals on Wheels users and referrers:

Likes – different lifestyles depicted; protective window over everyone; very attractive. Like the QR codes – takes you exactly where you need to go. Like the idea of lots of different people with different lifestyles, all under one roof. Suggest include a man as service user. Image of a person with temporary impairment/ disability (e.g. broken leg/ arm).

Dislikes – image that looks like a couple in the kitchen. Suggestion to include ethnic minority groups in this image.

Language – too many words on the page/ too wordy; heading MoWs could be a bit punchier (‘is this for you?’); don’t need to state ‘person referring’ or ‘community client’. Both columns of text repeat similar/ same messages – do we need to say it twice? Suggestion of pairing down the language. Too busy, too much on it. Needs to catch people’s eyes quickly. Spelling error: ‘Talk to you GP’ – suggestion that we need a phone number or a message like ‘Just ring your GP or social services’. Accompanied by a picture of a person on a phone. Informality of right hand column text is much more suitable.

Suggestion – change text to: **familiar** face, rather than ‘friendly face’.

Some discussion about conveying variety of foods and diversity – hard to convey different food types and dietary needs.

Colours – like it, subtle colour scheme. Not jumping out of your face.

General layout – protective roof; block of flats; like the cheeky sun peeping from the clouds. Suggestion of a delivery van with an image of hot soup. Suggest if there was only one column of images we could have bigger images of hot food.

Access – need easier access, not just a URL and QR code.

Suggestion for restructure: How can MoWs work for you? Simple messages. Suggestion of one message per line.

**Table S5:** Workshop 1 observation checklist


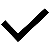
**Data Collection Checklist (one to be completed per workshop)**

1. Completed the observation template (this document)*
   **Main document which we will base the majority of our analysis on.*
2. Used the Zoom recording facility to capture group-based conversations

**Observation Template**

**Observer**:

**Time / date**:

**Number of attendees**:

There are five sections to this observation template, and notes should relate to: 1) the workshop activities; 2) the workshop as a whole; 3) the interaction between participants; 4) the workshop facilitation, and; 5) your reflections on the workshop. There is also space at the end of this document for any additional notes.

**Section 1: Workshop Activities**

Within the sections below, we are predominantly interested in capturing notes on the discussion that participants are having and their responses to the questions asked by the facilitator. Please make notes on the content of the discussion, and key phrases that are being used, and any additional interactions that you hear between participants.

***Activity 1* – Message content (i.e., what should go into the message)**

1.1 What are the terms in the findings’ themes and sub-themes that are unclear to the group?

1.2 What do they say are the reasons for themes/ sub-themes being unclear?

1.3 What should the *content of the message* be? (e.g. benefits of MoWs to service users, benefits to referrers, what should be improved about MoWs). Also consider the *formatting* of the message (e.g. length of message or personalised content).

For the infographics:

For the film:

1.4 What themes/ sub-themes should be incorporated in the knowledge translation tools?

For the infographics:

For the film:

1.5 Any additional conversation points around this?

**Section 2: Workshop as a whole**

2.1 Any additional notes and observations on the delivery and interaction with the workshop as a whole?

**Section 3: Interaction between attendees**

3.1 Do the participants seem to know each other prior to workshop? Any additional comments on this?

3.2 Any dominant individuals influencing the workshop? If so, what was the impact on the session?

3.3 Are participants getting the opportunity to talk/ express themselves? Any comments?

3.4 If issues around the group dynamic arose, how was this managed?

**Section 4: Facilitation**

4.1 Are there any key points to note about the facilitation of the workshop, and whether this influenced how participants engaged with the activities or the responses they provided?

**Section 5: Researcher reflections**

5.1 What went well within the workshop and why? (i.e. strengths)

5.2 What didn’t work too well and why? Can this be improved prior to the next workshop? (i.e. weaknesses)

5.3 Any other salient points of the workshop?

5.4 Did the presence of the research team have an impact on the workshop? If so, what impact or influence do you think you had?

**Table S6:** Workshop 2 observation checklist


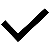


**Data Collection Checklist (one to be completed per workshop)**

1. Completed the observation template (this document)*
   **Main document which we will base the majority of our analysis on.*
2. Used the Zoom recording facility to capture group-based conversations

**Observation Template**

**Observer**:

**Time / date**:

**Number of attendees**:

There are five sections to this observation template, and notes should relate to: 1) the workshop activities; 2) the workshop as a whole; 3) the interaction between participants; 4) the workshop facilitation, and; 5) your reflections on the workshop. There is also space at the end of this document for any additional notes.

**Section 1: Workshop Activities**

Within the sections below, we are predominantly interested in capturing notes on the discussion that participants are having and their responses to the questions asked by the facilitator. Please make notes on the content of the discussion, and key phrases that are being used, and any additional interactions that you hear between participants.

***Activity* – Language (i.e., the terminology and tone) of the message**

Note, the purpose of the message should be to encourage people with care and support needs to access MoWs or encourage policymakers to enhance the service.

1.1 What should the actual messages be? Note, content may be positively or negatively framed (gains and losses) and may be generalised or customised to specific groups.

For the infographics:

For the film:

1.2 What should the language/ tone of the message be? Note, language may be positively or negatively framed (gains and losses) and may be generalised or customised to specific groups.

For the infographics:

For the film:

1.3 What should the films feature? (e.g. whose voices will be included/ represented, and the filming location).

1.5 Any additional conversation points around this?

**Section 2: Workshop as a whole**

2.1 Any additional notes and observations on the delivery and interaction with the workshop as a whole?

**Section 3: Interaction between attendees**

3.1 Do the participants seem to know each other prior to workshop? Any additional comments on this?

3.2 Any dominant individuals influencing the workshop? If so, what was the impact on the session?

3.3 Are participants getting the opportunity to talk/ express themselves? Any comments?

3.4 If issues around the group dynamic arose, how was this managed?

**Section 4: Facilitation**

4.1 Are there any key points to note about the facilitation of the workshop, and whether this influenced how participants engaged with the activities or the responses they provided?

**Section 5: Researcher reflections**

5.1 What went well within the workshop and why? (i.e. strengths)

5.2 What didn’t work too well and why? Can this be improved prior to the next workshop? (i.e. weaknesses)

5.3 Any other salient points of the workshop?

5.4 Did the presence of the research team have an impact on the workshop? If so, what impact or influence do you think you had?

**Table S7:** Workshop 3 observation checklist


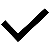
**Data Collection Checklist (one to be completed per workshop)**

1. Completed the observation template (this document)*
   **Main document which we will base the majority of our analysis on.*

2. Used the Zoom recording facility to capture group-based conversations

3. Participants are shown the infographics

**Observation Template**

**Observer**:

**Time / date**:

**Number of attendees**:

There are five sections to this observation template, and notes should relate to: 1) the workshop activities; 2) the workshop as a whole; 3) the interaction between participants; 4) the workshop facilitation, and; 5) your reflections on the workshop. There is also space at the end of this document for any additional notes.

**Section 1: Workshop Activities**

Within the sections below, we are predominantly interested in capturing notes on the discussion that participants are having and their responses to the questions asked by the facilitator. Please make notes on the content of the discussion, and key phrases that are being used, and any additional interactions that you hear between participants.

***Activity* – Feedback on the infographics**

Note, the purpose of the infographics should be to encourage people with care and support needs to access MoWs or encourage referrers to refer someone to the service.

1.1 Regarding the infographics, what are the reflections and comments from the group on these?

Infographic 1:

Note, for the following questions, ensure the discussion covers aspects of:

Acceptability of design; Layout; Visualisation of findings; Comprehensiveness of content and language; Balance between visualisation, images and text; Acceptability for different audiences; Reflection of the agreed messages

1.2 What aspects of the infographics do participants like, and why?

Infographic 1:

1.3 What are the aspects of the infographics that participants don’t like (or would like to see improved), and why?

Infographic 1:

1.5 Any additional conversation points around this?

**Section 2: Workshop as a whole**

2.1 Any additional notes and observations on the delivery and interaction with the workshop as a whole?

**Section 3: Interaction between attendees**

3.1 Do the participants seem to know each other prior to workshop? Any additional comments on this?

3.2 Any dominant individuals influencing the workshop? If so, what was the impact on the session?

3.3 Are participants getting the opportunity to talk/ express themselves? Any comments?

3.4 If issues around the group dynamic arose, how was this managed?

**Section 4: Facilitation**

4.1 Are there any key points to note about the facilitation of the workshop, and whether this influenced how participants engaged with the activities or the responses they provided?

**Section 5: Researcher reflections**

5.1 What went well within the workshop and why? (i.e. strengths)

5.2 What didn’t work too well and why? Can this be improved prior to the next workshop? (i.e. weaknesses)

5.3 Any other salient points of the workshop?

5.4 Did the presence of the research team have an impact on the workshop? If so, what impact or influence do you think you had?

**Table S8:** Workshop 4 observation checklist


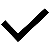
**Data Collection Checklist (one to be completed per workshop)**

1. Completed the observation template (this document)*
   **Main document which we will base the majority of our analysis on.*

2. Used the Zoom recording facility to capture group-based conversations

3. Participants are shown the final versions of the infographics and film to participants

**Observation Template**

**Observer**:

**Time / date**:

**Number of attendees**:

There are five sections to this observation template, and notes should relate to: 1) the workshop activities; 2) the workshop as a whole; 3) the interaction between participants; 4) the workshop facilitation; and 5) your reflections on the workshop. There is also space at the end of this document for any additional notes.

**Section 1: Workshop Activities**

Within the sections below, we are predominantly interested in capturing notes on the discussion that participants are having and their responses to the questions asked by the facilitator. Please make notes on the content of the discussion, and key phrases that are being used, and any additional interactions that you hear between participants.

***Activity* – Feedback on the infographics**

1.1 Regarding the infographics, what are the reflections and comments from the group on these?

Infographic for service users and referrers:

Infographic for commissioners and policy makers:

Note, for the following questions, ensure the discussion covers aspects of:

Acceptability of design; Layout; Visualisation of findings; Comprehensiveness of content and language; Balance between visualisation, images and text; Acceptability for different audiences; Reflection of the agreed messages

1.2 What aspects of the infographics do participants like, and why?

Infographic for service users and referrers:

Infographic for commissioners and policy makers:

1.3 What are the aspects of the infographics that participants don’t like (or would like to see improved), and why?

Infographic for service users and referrers:

Infographic for commissioners and policy makers:

1.5 Any additional conversation points around this?

***Activity* – Identifying the audiences and ways to share the infographics**

1.1 Who should be the target audience (e.g. GPs, MoWs managers, service users, charities…)?

For the infographics:

For the film:

1.2 How should the *message be communicated*? (i.e. what mechanisms and mediums should be used to share the message?). Consider the *mode of deliver*y (e.g. how will messages be shared, targeted at groups, in which settings, how often is message shared).

For the infographics:

For the film:

1.3 Any additional conversation points around this?

**Section 2: Workshop as a whole**

2.1 Any additional notes and observations on the delivery and interaction with the workshop as a whole?

**Section 3: Interaction between attendees**

3.1 Do the participants seem to know each other prior to workshop? Any additional comments on this?

3.2 Any dominant individuals influencing the workshop? If so, what was the impact on the session?

3.3 Are participant getting the opportunity to talk/ express themselves? Any comments?

3.4 If issues around the group dynamic arose, how was this managed?

**Section 4: Facilitation**

4.1 Are there any key points to note about the facilitation of the workshop, and whether this influenced how participants engaged with the activities or the responses they provided?

**Section 5: Researcher reflections**

5.1 What went well within the workshop and why? (i.e. strengths)

5.2 What didn’t work too well and why? Can this be improved prior to the next workshop? (i.e. weaknesses)

5.3 Any other salient points of the workshop?

5.4 Did the presence of the research team have an impact on the workshop? If so, what impact or influence do you think you had?
